# Regional trends and forecasting of under-five child mortality in Bangladesh: A mixed method approach of Functional ANOVA and Bayesian spatiotemporal modeling

**DOI:** 10.1371/journal.pone.0344934

**Published:** 2026-05-27

**Authors:** Md. Ismail Hossain, M. Sheikh Giash Uddin, Azizur Rahman, Shuvongkar Sarkar

**Affiliations:** 1 Department of Mathematics and Statistics, Mississippi State University, Mississippi State, Mississippi, United States of America; 2 Department of Statistics, Jagannath University, Dhaka, Bangladesh; 3 Department of Statistics and Data Science, Jahangirnagar University, Dhaka, Bangladesh; 4 Social Innovation Office, Department of Families, Government of Manitoba, Manitoba, Canada; 5 Department of Community Health Sciences, University of Manitoba, Manitoba, Canada; 6 Statistics Program, BRAC University, Dhaka, Bangladesh‌‌; VART Consulting PVT LTD, INDIA

## Abstract

**Background:**

This study aims to estimate historical under-five mortality rates from 1990 to 2022 across the eight divisions of Bangladesh, analyze regional disparities, and forecast under-five mortality rates trends up to 2030 considering both temporal and spatial dynamics.

**Methods:**

A cross-sectional study design was employed, utilizing nationally representative data from the Bangladesh Demographic and Health Survey 2022. The study included 64,697 records of children representing births from eligible women with full birth history across the eight divisions of Bangladesh. The dataset spans 33 years, enabling a detailed examination of under-five mortality rates trends at the sub-national level. The analysis included historical under-five mortality rates estimation and Functional ANOVA was conducted to explore regional disparities, while a Bayesian Spatiotemporal model was employed to forecast under-five mortality rates trends up to 2030. The permutation F-test was used to assess the statistical significance of the regional differences.

**Results:**

The permutation F test conclude that while regional differences in child mortality rates were not globally significant over the entire period from 1990 to 2022, significant disparities did emerge during specific years (early 1990s and late 2010s). Barishal, Rangpur, and Sylhet showed high variability in under-five mortality rates reductions in the early 1990s, while Dhaka and Khulna exhibited more consistent progress. ICAR spatial effects indicate that Sylhet and Rangpur, have slower reductions, highlighting persistent challenges in these areas despite national progress. Additionally, spatiotemporal modeling indicates all divisions in Bangladesh show significant reductions from 1990 to 2022, with Sylhet consistently having the highest rates and Khulna the lowest. Projections indicate a plateauing trend, with most divisions nearing the sustainable development goal target of 25 deaths per 1000 live births by 2030, though Sylhet and Rangpur may require additional interventions.

**Conclusions:**

Future strategies should focus on reinforcing healthcare infrastructure, enhancing maternal and child health services, and reducing socioeconomic inequalities to achieve faster improvements in high-mortality regions. Targeted interventions in these areas are necessary to achieve the SDG target by 2030, thereby improving child health outcomes nationwide.

## Introduction

The under-five mortality rate (U5MR) is a critical indicator of child health, representing the probability that a child will die before reaching five years of age, expressed per thousand live births [1]. Significant improvements in global public health have resulted in a two-thirds reduction in U5MR over recent decades, with rates declining from 93 per 1000 live births in the 1990s to 37 in 2020 [2–4]. However, the situation in Sub-Saharan Africa and South Asia remains alarming, with these regions accounting for approximately 80% of child deaths globally [5–7]. In 1990, 31% of global under-five deaths contributed by Sub-Saharan Africa, which has increased from to 55% by 2020, the highest among low- and middle-income countries [2]. In South Asia, one in every nineteen children dies before their fifth birthday, constituting nearly 30% of the world’s total child mortality [8].

Like other LMICs, under-five mortality is also a major public health problem in Bangladesh [9]. The 2017−18 Bangladesh Demographic and Health Survey (BDHS) data show that the rate of under-five mortality rate declined since 1990, and it performs about two-and-a-half times better than Pakistan (under-five mortality was 67 of thousand live births) and India is little behind Bangladesh which is 38 of 1000 live births [10–12]. Although Bangladesh has reduced under-five mortality at the national level in the past thirty years, the rate of decline at the sub-national level has not been significant at all. It has likewise seen that the Sylhet Division has the largest child mortality rate rather than the other region in Bangladesh [13].

Reducing U5MR is a crucial indicator of improved health services and reflects a country’s overall socioeconomic condition and quality of life [14]. The global community has committed to the Millennium Development Goals (MDGs), where reducing child mortality was a key target, and now towards the Sustainable Development Goals (SDGs), which aim to reduce U5MR to at least 25 deaths per 1000 live births by 2030 [15]. Achieving these goals necessitates effective strategies for allocating resources to reduce child health inequalities within countries, particularly focusing on areas most in need.

Proper diagnosis and measures to protect maternal and child health can significantly reduce U5MR risks. Although previous studies have explored risk factors for child mortality using count regression models [16], and have focused on national trends, there remains a gap in understanding sub-national variations in mortality rates and identifying specific regions that have experienced the most significant declines [17]. Additionally, it is crucial to examine the time periods during which significant regional differences are observable and to project future trends.

One approach to address this gap is Functional Data Analysis (FDA), a non-parametric method that expresses discrete time-series observations as functions. For instance, a recent study used FDA to model daily activity patterns in UK primary school-aged children [18]. Another study applied functional principal component analysis (FPCA) to describe growth trajectories of children under three years of age [19]. Additionally, FDA, particularly Functional Analysis of Variance (FANOVA), has enhanced predictions of influenza patterns in the US [20]. For future projections, employing the Bayesian spatiotemporal smoothing model is effective [21], which considers survey design effects on uncertainty in spatial and temporal estimates. This methodology also applied by [22] integrates both the survey design effects [21] and the conventional space-time random effects framework [23].

This study addresses the research questions: “How have mortality trends varied across divisions up until now?” and “How will these trends evolve in the future, considering both time and space?”. To fill these gaps, we estimated historical child mortality rates (1990–2022) across the eight divisions of Bangladesh using national representative survey data. The primary objective is to analyze spatiotemporal disparities in child mortality through functional analysis of variance (FANOVA). Additionally, we will forecast U5MR up to 2030 using a Bayesian spatiotemporal model, incorporating both temporal and spatial dynamics. This comprehensive analysis aims to inform targeted public health interventions designed to reduce child mortality in Bangladesh and also reduce differences across regions.

## Materials and methods

### Source of data, sample design, and sample size

This study used nationally representative cross-sectional data called the Bangladesh Demographic and Health Survey (BDHS), 2022. The United States Agency for International Development (USAID) in Bangladesh provided financial support for this investigation. The dataset is publicly available for research.

The BDHS, 2022 employed a two-stage stratified sampling method, as executed by the Bangladesh Demographic and Health Survey authority. The country was stratified by urban and rural areas within each of the eight administrative divisions of Bangladesh, resulting in 16 strata. Within each stratum, enumeration areas (EAs) from the 2011 Population and Housing Census were selected using probability proportional to size (PPS) sampling and a total of 675 EAs were selected in the first stage. In each selected EA, a complete household listing was conducted, and 45 households were selected systematically in the second stage. This study utilized the full birth history which provides detailed records for every child born to eligible women, allowing for the estimation of U5MR up to 33 years prior to the survey. For sub-national analysis, the study examined mortality rates across Bangladesh’s eight divisions, covering a 33-year period. The methodology applied follows the approach developed by [21] ensuring robust estimation of U5MR over distinct time periods and divisions. A total of 64,697 children’s records were included, enabling precise calculation of mortality trends at the divisional level. All analyses were conducted based on the available data from these 33 years across eight divisions. The code used for these estimations is available upon request from the author, ensuring transparency and reproducibility of the study’s outcomes.

### Basics of functional data

Assume that we have a real valued random process Y(t), which can be expressed as,


$$\begin{array}{c} y_{i}(t)=x_{i}(t)+\epsilon_{i}(t);where\;i=\text{1},\text{2},\ldots ,\;n;t=\text{1},\text{2},\ldots ,T \end{array}$$

Here, $y_{i}(t)$ represents the outcome, specifically the under-five child mortality rate, at time point t for division i. In this study, we consider 33 time points (from 1990 to 2022) across 8 sub-national regions/divisions of Bangladesh. Each division is treated as a sample (curves or functions), with each year serving as a designated time point. For a detailed explanation of basic functional data, refer to [20].

### Analysis of variance of functional data

In this study, we employed the Functional ANOVA (FANOVA) technique introduced by Ramsay and Silverman within the framework of functional regression analysis [24]. The response variable consisted of functions or curves, while the covariates comprised dummy variables representing categorical factors. FANOVA utilizes permutation tests for hypothesis testing, which involves reshuffling data labels to gene3rate a null distribution of the F statistic under the null hypothesis. By comparing the observed F statistic to this distribution, researchers can evaluate the significance of group differences without relying on strict distributional assumptions, making it a robust method for analyzing functional data. A recent study by Rahman and Jiang [20] provides a detailed explanation of FANOVA. FANOVA enable to compare entire mortality curve across divisions including differences in overall level, shape, and temporal evolution over the whole study period, rather than focus only discreate change points as traditional models.

### Bayesian spatiotemporal smoothing model

As we have said before, we followed the method developed by [21] to estimate the specific mortality rate in the sub-national period. After getting the place and time probability of death for different age groups, we use these as inputs to the spatiotemporal Bayesian hierarchical model to smooth the estimation across time and space. In this study, we adopt the final stage model, which is similar to the space-time model [23], that is, $\mu +\alpha_{t}+\gamma_{t}+\theta_{i}+\phi_{i}+\delta_{it}$. Here, $\alpha_{t}=unstructured\;time$, $\theta_{i}=unstructured\;space$, $\gamma_{t}=stuctured\;time$, $\phi_{i}=stuctured\;space$, and $\delta_{it}=space\;\&\;time\;interaction$. The two candidate models we consider are given below, with the random effects specification.


$$\begin{array}{c} Model\;I:\;\mu +\alpha_{t}+\gamma_{t(RW\text{1})}+\theta_{i}+\phi_{i}+\delta_{it} \end{array}$$

$$\begin{array}{c} Model\;II:\;\mu +\alpha_{t}+\gamma_{t(RW\text{2})}+\theta_{i}+\phi_{i}+\delta_{it} \end{array}$$

There are two temporal terms with $\alpha_{t}$ being independent and identically distributed random effects that pick up short-term fluctuations with no structure, and $\gamma_{t}$ be the random temporal terms (random walk prior of order 1 (RW1) for model I, and random walk prior of order 2 (RW2) for model II), to pick up local temporal smooth fluctuations, for t=1,…,T=33 time periods. The Intrinsic Conditional Autoregressive (ICAR) model was used to smooth the spatial term.

It is important to note that the structured temporal effect $\gamma_{t}$ modeled via RW2 produces smooth extrapolations beyond the observed period because the RW2 prior constrains second-order differences, penalizing abrupt changes in the trend’s acceleration. Within the historical window, the unstructured temporal term $\alpha_{t}$ absorbs short-term year-to-year fluctuations. However, since $\alpha_{t}$ carries no autoregressive structure, it is not projected forward; consequently, the 2022–2030 forecast reflects only the smooth RW2 component. This design choice prioritizes stable trend extrapolation consistent with the long-run trajectory, while the widening posterior credible intervals toward 2030 capture increasing uncertainty over time.

### Hyperpriors

For all random effects, we specify flat (weakly informative) priors on the precision τ, such that the 95% prior interval for residual odds rations lies in [0.5, 2], leading to Gamma(a,b) priors with a=0.5, b=0.001. This diffuse specification allows the observed 33-year divisional mortality data, rather than prior assumptions, to govern the degree of spatial and temporal smoothing, which is appropriate given the substantial heterogeneity in U5MR trends across Bangladesh’s eight divisions. This follows the convention established in Bayesian disease mapping [25,26], and is consistent with the empirical range of variation observed in the BDHS 2022 dataset. For this study we used gamma prior for the RW1 of $\tau_{\gamma }\sim gamma(0.5,\;0.001)$, for the RW2 of $\tau_{\gamma }\sim gamma(0.5,\;0.001)$, and for the ICAR of $\tau_{\phi }\sim gamma(0.5,\;0.001)$.

### Model selection

The main goal of Bayesian spatiotemporal approach was to compare and find the best random temporal prior for forecasting U5MR using BDHS, 2022 data. This used two approaches for comparing models,

i Logarithm of conditional predictive ordinate (LCPO)
ii Deviance information criterion (DIC)

A good model will have relatively high value of LCPO and a low value of DIC.

### Software

Model fitting was performed within the R programming computing environment, leveraging its advanced statistical capabilities. The “fda” package was employed to complete the Functional Analysis of Variance (FANOVA). For the Bayesian spatiotemporal models, the Integrated Nested Laplace Approximation (INLA) was utilized, as implemented in the “INLA” package. This approach provided robust and efficient estimation techniques suitable for the complex spatiotemporal data structure in this study.

## Results

### Smoothed functional data

**Fig 1:**
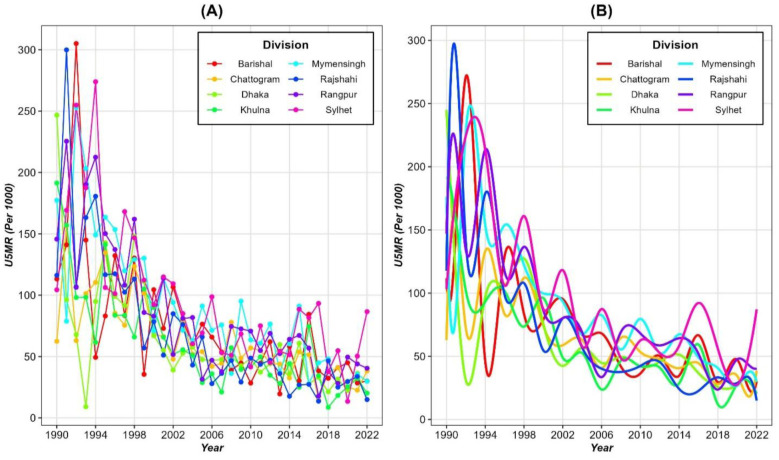
Trends in U5MR across divisions in Bangladesh (1990–2022): (A) Raw estimates and (B) Smoothed estimates of U5MR for each division.

Fig 1 illustrates the temporal trends in the U5MR per 1,000 live births across Bangladesh’s eight divisions from 1990 to 2022, using two different representations: panel (A) showing raw estimates, and panel (B) presenting smoothed estimates for each division. In Panel A, the raw U5MR data exhibit high variability, especially in the earlier years (1990–1995), reflecting wide disparities in child survival between divisions. The rates decline significantly over time. However in Rangpur and Sylhet division mortality rates remained relatively high for extended periods. The variability diminishes from 2000 onwards, corresponding to broader national efforts to improve child health, though regional disparities are still noticeable. Panel B provides a smoothed representation of U5MR trends, reducing noise in the data and highlighting more general patterns across divisions. As the data nature was non-periodic, we applied B-spline basis functions in this case. From panel B of Fig 1, a steep decline in U5MR between 1990 and 2000 is evident across all divisions. However, the persistent higher mortality in divisions such as Rangpur and Sylhet indicate the need for targeted interventions in these regions. Although most divisions show convergence in U5MR by 2022, Sylhet and Rangpur continue to display elevated mortality rates relative to the national average, highlighting regional inequities in child survival.

### Mean smoothing curve

**Fig 2:**
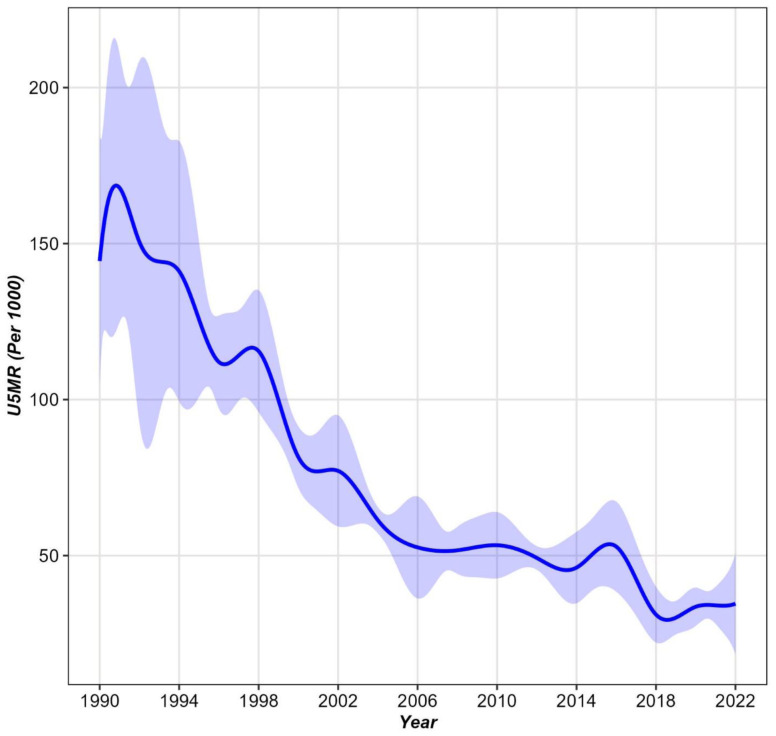
Mean smoothing curve with 95% CI of U5MR in Bangladesh (1990–2022).

Fig 2 depicts the mean smoothing curve of the U5MR per 1,000 live births in Bangladesh between 1990 and 2022, with shaded 95% confidence intervals to indicate uncertainty around the estimates. The curve reveals a steep decline in U5MR over the observed period, particularly from 1990 to 2005. After 2005, the decline in U5MR continued at a slower, more stable pace, with smaller fluctuations observed between 2010 and 2022. The slight uptick in U5MR around 2018 could be indicative of temporary public health challenges or disparities in access to care across different regions, but the overall downward trend suggests sustained progress in reducing child mortality over the past three decades. The confidence intervals were widest in the early 1990s, reflecting higher variability in mortality outcomes across different regions and population groups at the time. Over time, the narrowing confidence intervals signal reduced regional disparities and a more uniform decline in U5MR across the country.

### Divisional differences in temporal patterns of U5MR

**Fig 3:**
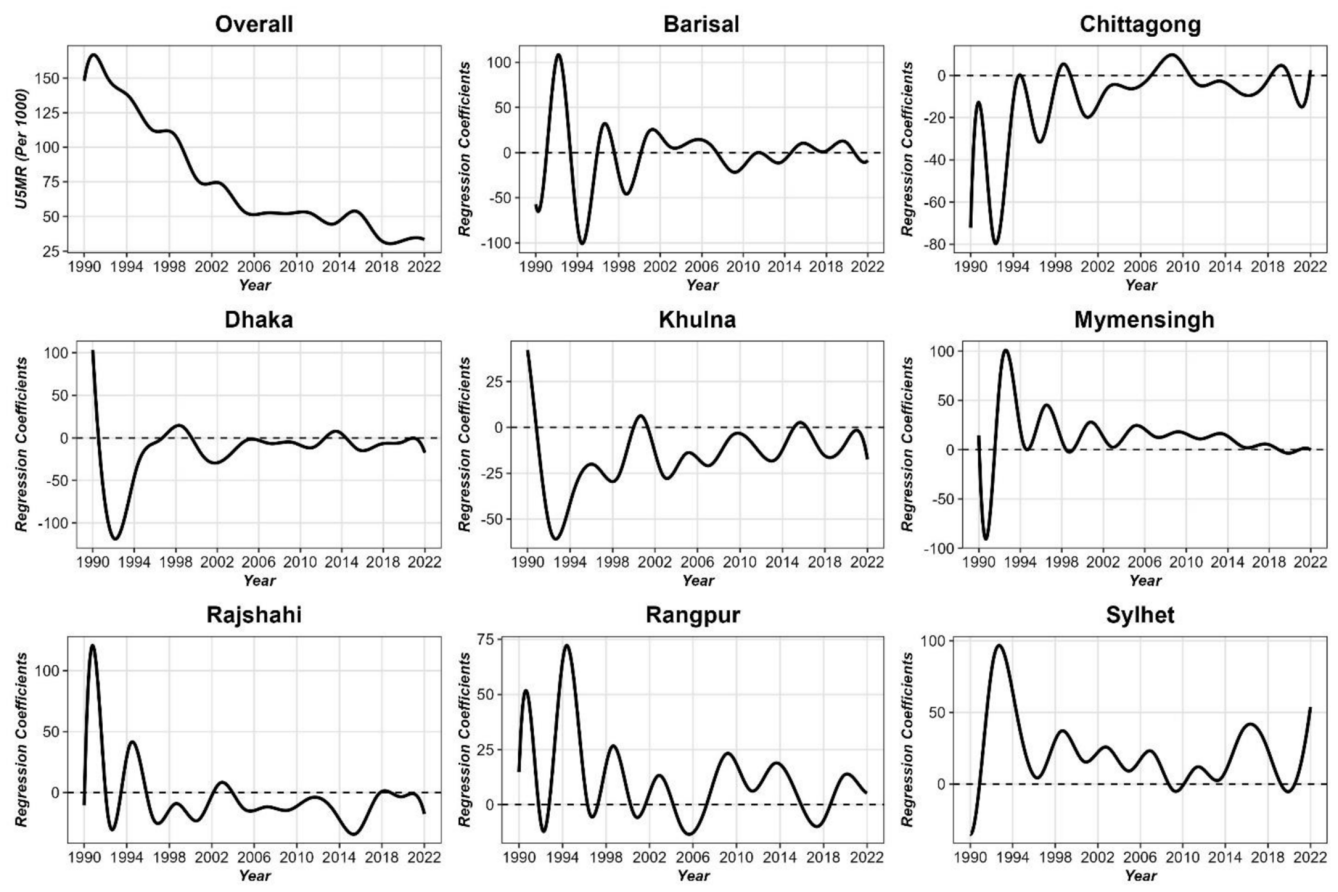
Functional ANOVA of U5MR coefficients by division in Bangladesh (1990–2022).

A one-way functional analysis of variance (FANOVA) was conducted to examine the divisional differences in temporal patterns of U5MR in Bangladesh. Fig 3 presents the results of the FANOVA conducted on the U5MR across eight divisions in Bangladesh from 1990 to 2022. The overall trend (top left) shows a consistent decline in U5MR, aligning with national averages, while the regression coefficients for each division (right panels) indicate regional variability in the magnitude and timing of these changes.

The regression coefficients demonstrate fluctuations over time, with divisions such as Barishal, Rangpur, and Sylhet exhibiting the most pronounced variability, especially in the early 1990s. In contrast, divisions like Dhaka and Khulna display more stable patterns with fewer extreme deviations, suggesting relatively more consistent progress in these regions. For divisions like Chattogram and Rajshahi, the regression coefficients indicate substantial early improvements, followed by periods of stagnation or slower declines post-2005. Mymensingh, a relatively newer division, shows modest improvements with more uniform reductions post-2000.

**Fig 4:**
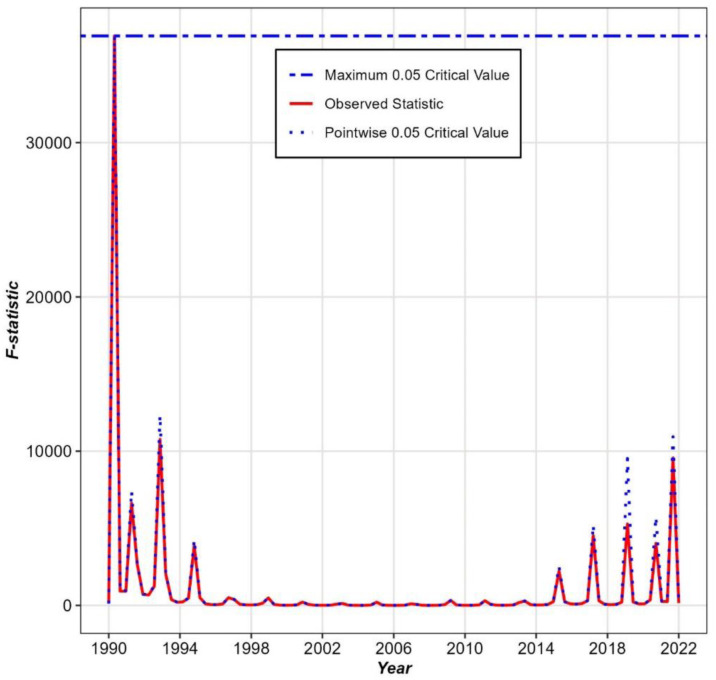
Permutation F test of Functional ANOVA.

Fig 4 represents a permutation F-statistic over time, a key output from the FANOVA. Red line shows the observed F-statistic at each time point (year). The F-statistic tests whether there are significant differences between divisions in the U5MR. Peaks in the observed statistic indicate times when differences between divisions were most pronounced. The maximum critical value is the threshold that the observed F-statistic must exceed for the differences to be considered statistically significant at the global level (across all time points). In this figure, the observed F-statistic (red) stays consistently below the maximum critical value (blue), indicating no global significance.

The pointwise critical value indicates the 0.05 significance threshold for individual time points. Some of the observed F-statistic values (red) exceed this pointwise threshold in specific years, particularly around 1990–1994 and 2018–2022. This suggests significant differences in child mortality across divisions during these periods, but only at certain time points, not consistently over the entire timeline. It is observed that global significance was observed, indicating that when averaged over the full study period, regional trajectories do not differ statistically. However, pointwise and interval-specific contrasts identify significant disparities during particular time periods, reflecting time-localized regional heterogeneity

### Bayesian spatiotemporal model

This study compares two candidate models, Model I (ICAR & RW1) and Model II (ICAR & RW2), using the LCPO and DIC as model evaluation metrics. Model I have a DIC value of 212.43 and an LCPO value of −110.66, while Model II has a DIC of 211.68 and an LCPO of −110.35. A good model is indicated by a lower DIC and a higher LCPO value. Based on both criteria, Model II is preferred over Model I.

### Summarization of variance components

Table 1 summarizes the variance components of Model II and their contributions to total variation. The structured spatial effect (ICAR space) explains 82% of the variation, indicating strong spatial autocorrelation. The unstructured spatial effect accounts for approximately 11%, showing substantial spatial heterogeneity. The unstructured temporal effect contributes 1.4%, and the structured temporal effect explains only 0.7%, indicating modest temporal structure. The space-time interaction term accounts for 5%, suggesting a minor interaction effect. According to variance analysis, spatial effects dominate the variation, with significant spatial autocorrelation and heterogeneity. Temporal effects are present but less significant, and the space-time interaction is minimal. This finding suggests that most disparities in child mortality are accounted for by spatial effects. So policymakers should focus on spatially targeted strategies, rather than uniform interventions across all regions

**Table 1:** Summaries of variance components.

*Variance*	*Interpretation*		*Percentage variation*
$\overset{2}{\underset{\theta }{\sigma }}$	Space unstructured	(Independent space)	10.7
$\overset{2}{\underset{\varphi }{s}}$	Space structured	(ICAR space)	82.2
$\overset{2}{\underset{\gamma }{s}}$	Time structured	(RW2 time)	0.7
$\overset{2}{\underset{\alpha }{\sigma }}$	Time unstructured	(Independent time)	1.4
$\overset{2}{\underset{\delta }{\sigma }}$	Space-time interaction	(Independent space-time interaction)	5.0

### Posterior median of spatial and temporal random effects

**Fig 5:**
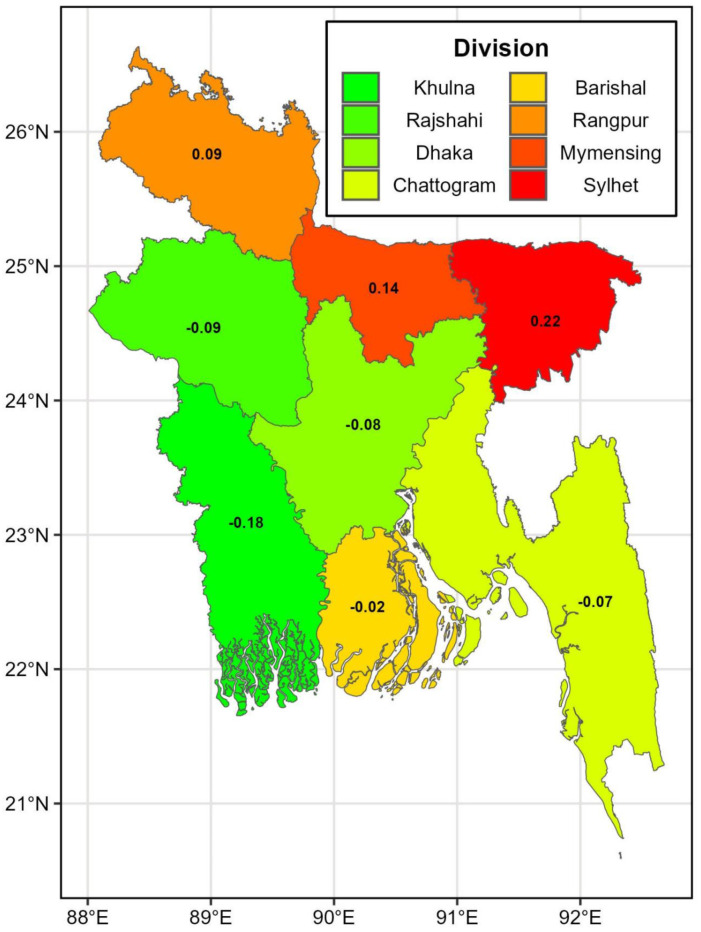
Posterior median of ICAR random effects $\left(\phi_{i}\right)$. Posterior median estimates of ICAR spatial random effects across administrative regions of Bangladesh. Administrative boundary shapefiles were obtained from https://data.humdata.org/dataset/cod-ab-bgd. The figure was generated by the authors using R software (sf and ggplot2 packages).

Fig 5 presents the posterior median estimates of the spatial random effect, modeled using the Intrinsic Conditional Auto-Regressive (ICAR) component, following a Bayesian spatiotemporal analysis of U5MR across divisions in Bangladesh. The map highlights the division-level variation in the ICAR effect, adjusting for spatial dependencies among neighboring divisions. The positive values observed in Sylhet (0.22) and Mymensingh (0.14) suggest that these divisions experienced higher-than-expected U5MR, even after accounting for temporal trends and covariates in the model. In contrast, the negative values in divisions such as Khulna (−0.18), Chattogram (−0.07), and Rangpur (−0.09) indicate that these regions had lower-than-expected mortality rates relative to the national average. This spatial heterogeneity underscores the importance of geographically tailored interventions. The elevated U5MR in Sylhet and Mymensingh, as captured by the positive ICAR random effects, suggests potential gaps in health services or socio-environmental factors that need to be addressed. Conversely, the improved performance in divisions with negative ICAR effects, like Khulna and Rangpur, highlights regions where successful health strategies may serve as models for other areas. Overall, the ICAR spatial effect indicates significant variation in under-5 mortality risk across the country, emphasizing the role of location-specific factors in shaping child survival outcomes.

**Fig 6:**
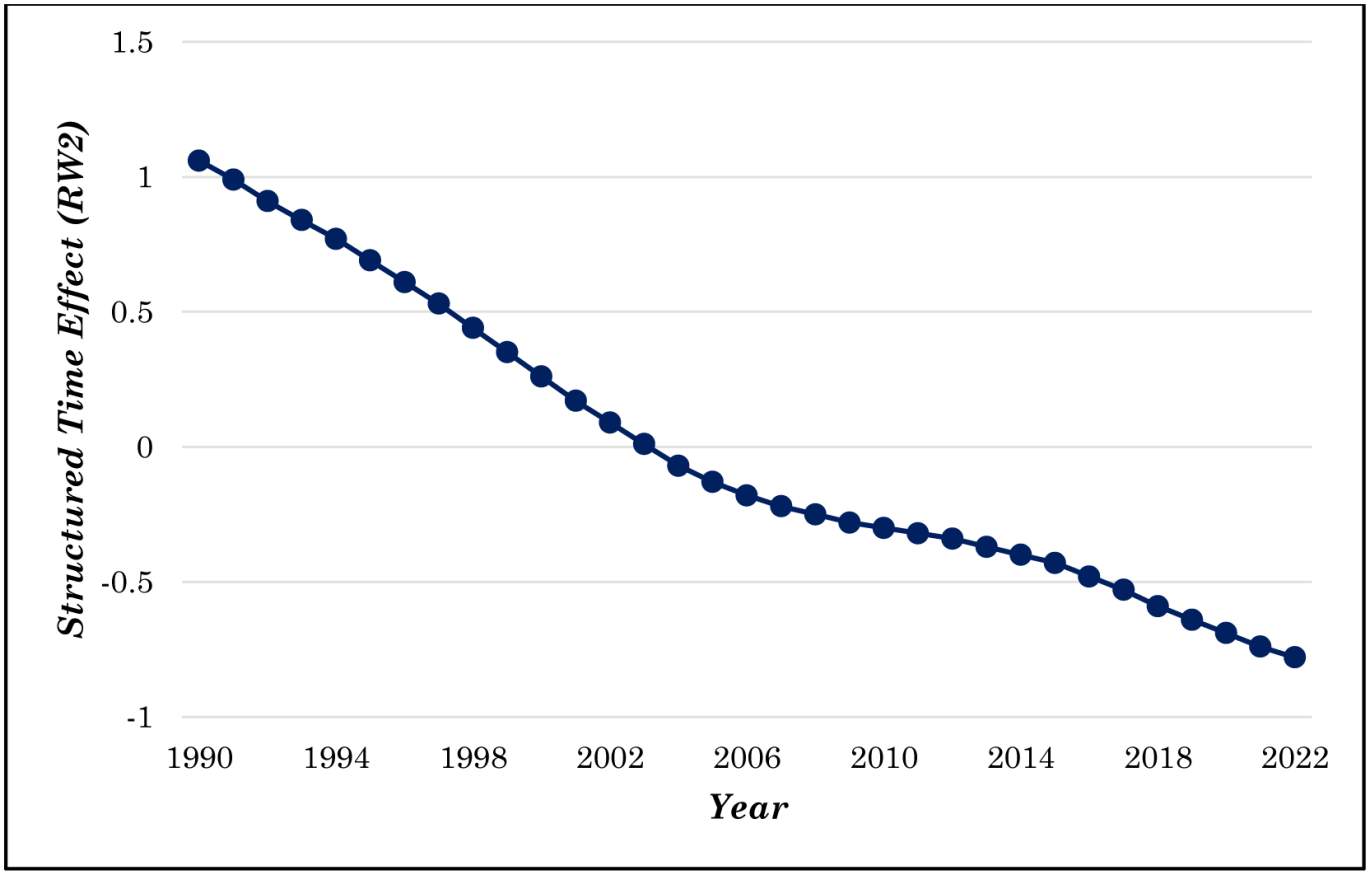
Posterior median of RW2 random effects $\left(\gamma_{t}\right)$.

Fig 6 illustrates the posterior medians of the structured temporal effect, modeled using a Random Walk of Order 2 (RW2), from the Bayesian spatiotemporal analysis of U5MR in Bangladesh. The RW2 captures the smoothed temporal trend, accounting for the structured evolution of U5MR over time. The structured time effect reveals a consistent decline from 1990 through 2022. In 1990, the RW2 effect starts above 1, signifying elevated U5MR, but gradually decreases, falling below zero by 2006. By 2022, the RW2 effect approaches −1, highlighting the dramatic reduction in U5MR over the last three decades. Overall, the RW2 temporal effect demonstrates the profound influence of time on the under-5 mortality trend in Bangladesh, with a substantial decline achieved over the past three decades, although additional efforts are necessary to sustain and accelerate this progress moving forward.

### Future projection of U5MR

**Fig 7:**
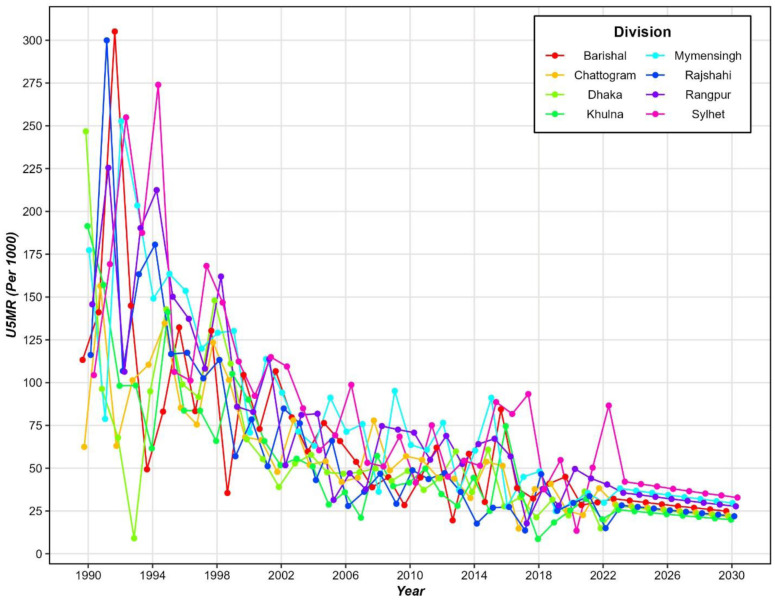
Division wise future projection of under-five mortality rates in Bangladesh.

Fig 7 presents the division-wise future projections of the U5MR in Bangladesh, using Bayesian spatiotemporal model (ICAR & RW2 random effects). The projections extend to 2030 and are compared against the Sustainable Development Goal (SDG) target of reducing U5MR to 25 deaths per 1000 live births by 2030. Each line represents a distinct division of Bangladesh, showing both the historical U5MR from 1990 to 2022 and future estimates up to 2030. The analysis highlights a significant reduction in U5MR across all divisions, with steep declines observed between 1990 and 2010. The division of Sylhet (magenta) consistently exhibited the highest U5MR in the earlier years, while Khulna (green) maintained the lowest rates throughout much of the time period. The projections for the upcoming years, from 2022 to 2030, show a plateauing trend, with most divisions expected to hover close to the SDG target of 25 deaths per 1000 live births. Barishal, Dhaka, and Khulna divisions demonstrate the most favorable trends, approaching or even surpassing the SDG target before 2030. In contrast, Sylhet and Rangpur show slower reductions and might need targeted interventions to achieve the SDG goal.

## Discussion

This study provides a comprehensive analysis of U5MR across the eight divisions of Bangladesh over a 33-year period (1990–2022), using data from the full birth history file of the BDHS 2022 dataset. FANOVA is applied for examining the effects of division-level disparities on child mortality, emphasizing regional factors influencing health outcomes and applying Bayesian spatiotemporal models, we estimated sub-national mortality rates, enabling us to capture both spatial and temporal trends in U5MR and provide forecasts up to 2030.

The analysis revealed a remarkable reduction in U5MR across all divisions, with a more pronounced decline observed between 1990 and 2010. However, significant variability in the pace of this reduction was noted among divisions. For example, Sylhet division consistently exhibited the highest U5MR rates in the earlier years, whereas Khulna demonstrated the most favorable trends, with the lowest rates for much of the study period. This heterogeneity suggests unequal access to and distribution of healthcare services, interventions, and socioeconomic improvements across regions, as noted in a recent study [27].

The FANOVA was performed to assess divisional differences in U5MR trends across Bangladesh from 1990 to 2022. Our divisional analysis highlighted important regional disparities in U5MR trajectories. Dhaka and Khulna exhibited relatively stable and consistent reductions, while Sylhet and Rangpur displayed more erratic patterns, particularly in the earlier decades. This disparity was observed among these divisions, likely due to misconceptions and lower awareness about child and maternal healthcare [13]. Sociodemographic and health factors like skilled birth attendance, lower proportion of 4 + ANC visits, Shortages of skilled health personnel, uneven facility readiness across regions, wealth and education contribute for higher U5MR for Sylhet and Rangpur [11,28]. Sylhet division also lags behind others in antenatal care, medically assisted deliveries, and child vaccination coverage due to affected by flash floor consistently [13,29]. Chattogram and Rajshahi showed initial rapid improvements, followed by stagnation in the post-2005 period. A study revealed associated factors for hill tracts of Chattogram division and suggested a need for sustained and renewed public health efforts [30]. The permutation F-statistic revealed no global significance for differences across divisions but identified significant disparities in specific periods, notably from 1990–1994 and 2018–2022.

By leveraging both intrinsic conditional autoregressive (ICAR) and random-walk second-order (RW2) models, the study provided insights into both structured time and spatial effects. The RW2 temporal effects showed a steady decline in mortality rates, aligning with overall national trends in child health improvements. Nonetheless, the ICAR spatial effects indicate that some divisions, such as Sylhet and Rangpur, have slower reductions, reflecting persistent challenges in these areas despite national progress. The projections for the period 2022–2030 suggest that while most divisions are on track to meet the Sustainable Development Goal (SDG) target of reducing U5MR to 25 deaths per 1000 live births by 2030, certain regions, notably Sylhet and Rangpur, may struggle to achieve this goal without intensified interventions. Sylhet’s consistently higher U5MR and slower reduction rates signal an urgent need for targeted healthcare programs, particularly those aimed at improving maternal and child health services, enhancing nutritional interventions, and increasing immunization coverage.

## Strengths and limitations

This study leverages a powerful combination of Functional Analysis of Variance (FANOVA) and Bayesian spatiotemporal modeling to provide nuanced insights into the historical and projected trends of under-five mortality across the eight divisions of Bangladesh. By applying FANOVA, we were able to simplify the detection of significant temporal and regional disparities, effectively highlighting divisions that experienced the most pronounced fluctuations in child mortality over the past three decades. The Bayesian spatiotemporal framework further strengthened the analysis by incorporating spatial dependencies and uncertainty in the modeling process, allowing for more reliable forecasts up to 2030. Additionally, the use of a comprehensive dataset covering 33 years of birth history records from nationally representative surveys enhanced the validity and depth of the findings.

Despite these strengths, some limitations must be acknowledged. One key limitation lies in the potential recall bias associated with retrospective survey data, as birth history information is self-reported and may be subject to inaccuracies. Furthermore, the precision of estimates may vary across regions, particularly in remote or underdeveloped divisions where sample sizes are smaller or where data collection may have been less consistent over time. These issues introduce a degree of uncertainty in trend estimation and forecasting for certain areas. Additionally, the smoothness of the 2022–2030 projection in Fig 7 is an inherent property of the RW2 prior, which extrapolates the long-run temporal trend while suppressing short-term fluctuations that are visible within the historical data. This implies that the point projections reflect a “smooth continuation” scenario and may not capture potential structural breaks, sudden policy changes, or epidemiological shocks that could produce non-smooth trajectories post-2022. Readers should interpret the projections as conditional forecasts under the current trend, with the widening credible intervals reflecting genuine uncertainty about the future pace of U5MR decline. A further limitation is that the Bayesian spatiotemporal model employed in this study does not explicitly incorporate time-varying covariates such as wealth index, maternal education, skilled birth attendance, antenatal care utilization, or vaccination coverage. The model was intentionally specified as a smoothing and forecasting framework following Mercer et al. [21], where the primary goal is reliable sub-national estimation under complex survey designs, rather than covariate-adjusted causal inference. The structured spatial effect (ICAR) implicitly captures division-level heterogeneity attributable to such socioeconomic and healthcare factors. Future work should integrate time-varying predictors within a joint modeling framework to disentangle the direct effects of health system investments from residual spatial heterogeneity.

## Conclusion

This study underscores the significant strides made in reducing U5MR across Bangladesh but also highlights the persistent disparities between divisions. Divisions like Khulna, Dhaka, and Barishal are on track to meet or even exceed SDG targets, while Sylhet and Rangpur lag behind. These findings suggest that while national-level policies have been effective, targeted interventions are essential to address regional disparities and ensure equitable progress towards child survival across Bangladesh. Future public health strategies should focus on strengthening healthcare infrastructure, increasing accessibility to maternal and child health services, and addressing socioeconomic inequalities to accelerate progress in high-mortality regions. While projections to 2030 suggest potential achievement of national SDG targets, depend on the continuing unchanged; unforeseen changes in nationwide health policies. Continued efforts will be essential to meet the SDG targets by 2030, with a particular focus on the divisions identified as high-risk in this analysis.
